# A method for reproducible high‐resolution imaging of 3D cancer cell spheroids

**DOI:** 10.1111/jmi.13169

**Published:** 2023-01-26

**Authors:** Thomas A. Phillips, Valeria Caprettini, Nandini Aggarwal, Stefania Marcotti, Rob Tetley, Yanlan Mao, Tanya Shaw, Ciro Chiappini, Maddy Parsons, Susan Cox

**Affiliations:** ^1^ Randall Centre for Cell and Molecular Biophysics King's College London London UK; ^2^ Centre for Craniofacial & Regenerative Biology King's College London London UK; ^3^ Department of Inflammation Biology School of Immunology & Microbial Sciences King's College London London UK; ^4^ Laboratory for Molecular Cell Biology and Institute for the Physics of Living Systems University College London London UK

**Keywords:** 3D bioimaging, cancer spheroids, cytoskeleton, hydrogel scaffold, structured illumination microscopy

## Abstract

Multicellular tumour cell spheroids embedded within three‐dimensional (3D) hydrogels or extracellular matrices (ECM) are widely used as models to study cancer growth and invasion. Standard methods to embed spheroids in 3D matrices result in random placement in space which limits the use of inverted fluorescence microscopy techniques, and thus the resolution that can be achieved to image molecular detail within the intact spheroid. Here, we leverage UV photolithography to microfabricate PDMS (polydimethylsiloxane) stamps that allow for generation of high‐content, reproducible well‐like structures in multiple different imaging chambers. Addition of multicellular tumour spheroids into stamped collagen structures allows for precise positioning of spheroids in 3D space for reproducible high‐/super‐resolution imaging. Embedded spheroids can be imaged live or fixed and are amenable to immunostaining, allowing for greater flexibility of experimental approaches. We describe the use of these spheroid imaging chambers to analyse cell invasion, cell–ECM interaction, ECM alignment, force‐dependent intracellular protein dynamics and extension of fine actin‐based protrusions with a variety of commonly used inverted microscope platforms. This method enables reproducible, high‐/super‐resolution live imaging of multiple tumour spheroids, that can be potentially extended to visualise organoids and other more complex 3D in vitro systems.

## LAY DESCRIPTION

Small groups of cancer cells can be embedded into a 3D scaffold to mimic cancer invasion seen in patients. Microscopy is a valuable tool for analysing cancer cell invasion in these models but is often limited by the level of detail achievable. Here, we present a method to achieve enhanced imaging of these processes by gaining control of the positioning of these cancer cell groups. This will improve understanding of the details of how cancer cells invade.

## 1 INTRODUCTION

Optical microscopy is the most widely used imaging technique due to general ease of use and relatively inexpensive equipment for the more basic instruments.^1^ Fluorescence microscopy, where specific proteins on the sample are labelled with molecules (fluorophores) that absorb light at one wavelength and emit at another, is highly popular. This is due to its ability to image the distribution of specific proteins, and because the output is a simple image of the sample as a signal on a dark background (in contrast to methods such as phase contrast, where the image generation process is complex). In fluorescence microscopy, samples are often imaged on inverted setups, where both the illumination and collection occur from below the samples. Such geometry is ideally suited to imaging optically transparent 2D samples such as single cells on a coverslip.

However, many biological samples are considerably thicker and 3D requiring alternative solutions that might involve lowering spatial and or temporal resolution, using phototoxic reagents or molecules, adding complexity to the sample preparation and so on. Several commonly employed imaging techniques share pros and cons in their use for imaging of 3D biological samples. Epifluorescence microscopy allows for excitation of fluorophores with an LED, mercury or xenon light source of a particular wavelength, resulting in emission of red‐shifted photons, with light collection via charge‐couple device (CCD) or scientific CMOS (sCMOS) camera.^2^ The simplicity and relatively low cost of epifluorescence instruments is coupled with comparatively inferior resolution and a lack of sectioning capability compared to more advanced imaging systems, rendering this technique unsuitable for thicker samples. The most common approach to achieve optical sectioning is point‐scanning confocal microscopy,^3^ which is an alternative approach wherein laser illumination improves illumination uniformity and a variable diameter pinhole proximal to the detector rejects out‐of‐focus light. This creates a sectioned image, allowing for high‐resolution 3D reconstructions of complex samples. However, the point‐scanning nature of this modality results in slow imaging with increased photo‐toxicity and bleaching which is particularly important when imaging live samples. Spinning disk confocal microscopy is perhaps the most common approach to overcome the slow speed of point‐scanning confocal microscopy.^4^ Instead of scanning the sample with a single focused point, an array of points is created using disks with regularly spaced pinholes (and, in a lower set of disks, micro‐lenses) in a spiral pattern (Nipkow disks). By sweeping an array of points over the sample, imaging is faster than point‐scanning confocal and less photo‐toxic, while still achieving sectioning. All the methods described here are limited by the Abbe limit, which quantifies how resolution is limited by the diffractive properties of light, for fluorescence microscopy in practice to around 200 nm.^5^

Photon reassignment (either computational or physical) is a recent innovation in confocal microscopy design that boosts the achieved resolution to, in principle, around 140 nm. A spinning‐disk‐based implementation of this concept resulted in the SoRa (Super‐resolution by Optical Reassignment) microscope, which leverages a second matched array of micro‐lenses to enable super‐resolution spinning disk imaging.^6^ While achieving higher resolution, the SoRa is still subject to the two major limitations of spinning disk techniques: increased background signal with dense samples due to pinhole crosstalk, and potential image artefacts caused by a mismatch between the disk spin and camera frame rate.

An alternative fluorescence technique employs two‐/multiphoton excitation, allowing for a nonlinear imaging modality that excels at deep sample imaging due to reduced scatter and background associated with simultaneous long wavelength excitation.^7^ The long wavelength, high‐intensity laser illumination also allows second harmonic generation imaging of unlabelled structures, as well as laser ablation due to high laser power. This extends the utility of multiphoton microscopy beyond imaging of labelled structures and means it can be particularly useful for label‐free imaging and manipulation of the ECM surrounding cancer cells. However, two‐/multiphoton imaging set‐ups are generally more complex and expensive than plain fluorescence techniques, single point‐scanning leads to long acquisition times, and high laser power requirements can often generate high temperatures making challenging long‐term or high temporal resolution imaging.

A commonly used approach for studying cancer cell invasion in vitro is the formation of multicellular tumour spheroids by hanging drop^8^ or ultra‐low attachment well plates^9^ followed by embedding within collagen or more complex matrices (e.g. basement membrane mimics such as Matrigel). The resulting samples represent a scenario which is more physiologically relevant than 2D models, while leveraging experimental flexibility and ease of use to circumnavigate complexities encountered when using organoids or in vivo mouse models. Embedding spheroids within collagen gels essentially involves pipetting liquid collagen into the well of a suitable plate, followed by manual addition of the spheroid and polymerisation of the collagen gel.^10^

While this approach can be suitable for bulk assessment of spheroid invasion, it can lead to highly inconsistent sample preparation within and between experiments^11^ and it may not be well suited to high‐ or super‐resolution imaging. Accurate and consistent positioning of spheroids in the Z‐plane within a volume of liquid collagen gel is challenging due in part to timing of collagen polymerisation. This results in most spheroids being too far away to image and there are particular problems associated with the use of short working distance high‐powered objective lenses meaning that consistent high‐resolution imaging of embedded spheroids is very challenging. Imaging of spheroids is additionally complicated by their density, with variability of spheroid positioning in the Z‐plane compromising consistency of spheroid invasion assays when imaging with confocal systems and higher‐magnification/numerical aperture (NA) objectives. Many advanced microscopy techniques rely heavily on accurate and consistent positioning of samples within the Z‐plane, with inaccurate positioning essentially nullifying the efficiency and benefits of these methods due to the working distance of objective lenses. Furthermore, inaccurate placement of spheroids can lead to settling of the spheroid on the coverslip, resulting in cells adopting 2D phenotypes, which is not reflective of in vivo invasion. Previous work from the Nelson lab^12^ led to accurate seeding of small organoids in 3D collagen gels by stamping gels with PDMS stamps. Here we describe the development of a user‐friendly spheroid invasion assay inspired by this previous work from the Nelson lab^12^ that has been optimised for high‐ and super‐resolution spheroid imaging and imaging of cell–ECM dynamics.

## 2 MATERIALS AND METHODS

### 2.1 Cell lines

MCF7 human breast carcinoma cells (ATCC) were grown in EMEM (Gibco); HCC1954 human breast carcinoma cells (ATCC) were grown in RPMI‐1640 medium (Sigma‐Aldrich) while HEK293T cells (ATCC) and normal dermal fibroblasts (NDFs) were both grown in high glucose DMEM (Gibco). All were supplemented with 10% v/v heat‐inactivated FBS (to inactivate residual complement proteins, Gibco), 2 mM L‐Glutamine (Gibco), 100 units/ml penicillin and 10 μg/ml streptomycin (Sigma‐Aldrich). NDF medium was additionally supplemented with 1% sodium pyruvate (Gibco). All cell lines were maintained in a sterile tissue culture incubator at 37˚C with a humidified 5% CO_2_ atmosphere. Cells were passaged at 80–90% confluency by washing once with PBS (calcium‐ and magnesium‐free) and passaged using trypsin in EDTA (0.05% concentration, Sigma‐Aldrich) at 37˚C. All cell lines were tested mycoplasma at least every two months via PCR using custom primers, with positive samples being destroyed.

### 2.2 Generation of stable cell lines

MCF7 cells were transiently transfected with GFP empty vector or GFP‐YAP using Lipofectamine 3000 (Thermo Fisher Scientific) according to manufacturer guidelines. To generate stable expression of transient constructs, cells were selected using 0.88 mg/ml of G418 (Roche) to maintain construct expression. To generate lentivirus containing lentiviral constructs, HEK293T cells were cotransfected with GFP‐lifeact lentiviral vector, p∆8.91 and envelope plasmids using PEI transfection reagent in Opti‐MEM (Gibco). DNA‐PEI mixture was added to HEK293T cells for 6 h at 37˚C. Medium was then changed to Opti‐MEM and cells were incubated for 48 h. Lentivirus was collected by gathering media, centrifuging at 2000RPM for 5 min then filtering with 0.22 μm filter to remove cell debris. Virus was used fresh to infect target cells.

### 2.3 Design and microfabrication of SU‐8 2100 master wafers

To microfabricate SU‐8 2100 (MicroChem) master wafers for subsequent creation of polydimethylsiloxane (PDMS) stamps, custom designed photomasks were printed on Agfa Idealine HPF (High Resolution Plotter Film) 0.18 mm thick polyester film at 5 μm line resolution (JD photodata UK). Photomasks used include 75 μm dots at 500 μm pitch, 100 μm dots at 500 μm pitch, 150 μm dots at 750 μm pitch and 200 μm dots at 950 μm pitch. SU‐8 2100 master wafers were generated using 100 mM diameter, single‐side polished silicon wafer (test grade, 500 μm thick, p‐type, resistivity 0–100 Ohm/cm, University Wafer inc.). All microfabrication work was carried out in a dust‐free, ventilated microfabrication lab. Silicon wafers were cleaned in an oxygen plasma cleaner (Zepto W6 Plasma Cleaner, Diener) set to 100 W power for 5 min and subsequently dehydrated at 200˚C for 30 min. The wafer was then spin‐coated with 10 ml SU‐8 2100 (Microchem) at 3000 RPM or 1500 RPM to generate 100 μm or 200 μm thick layers, respectively. Wafers spin coated with SU‐8 2100 were then soft baked first at 65˚C for 5 min then at 95˚C for 12 min (100 μm thick) or 15 min (200 μm thick). The substrate was UV exposed through the desired photomask using a UV‐KUB2 exposure masking system (KLOÉ) at 35 mW/cm^2^ for 15 s (100 μm) or 35 s (200 μm). Following UV irradiation, postexposure baking of master wafers was carried out for 5 min at 65˚C followed by further baking for 12 min (100 μm) or 15 min (200 μm) at 95˚C. SU‐8 2100 masters were then developed by submersing in SU‐8 developer (Microchem) for 20 min on a plate shaker. Masters were then dried and reflowed for 1 min at 130˚C before clean storage until microfabrication of PDMS stamps.

### 2.4 Microfabrication of PDMS stamps

Prior to microfabrication of PDMS stamps, the SU‐8 2100 masters, feature‐side up, were exposed to oxygen plasma at 100 W power for 10 min. Masters were then functionalised with trichloro(1H,1H,2H,2H‐perfluorooctyl)silane (Sigma‐Aldrich) by gas phase in a desiccator for 1 h. Silanisation served to promote the detachment of PDMS from the SU‐8 2100 master upon demoulding. Following silanisation, SU‐8 2100 masters were washed twice with acetone, once with isopropanol and dried under N_2_ flow. For casting of PDMS polymer onto SU‐8 2100 masters, a 10:1 Sylgard 184 Elastomer (PDMS) and curing agent mix (w/w, Dow Chemicals) was made up and degassed for at least 30 min in a desiccator until all visible air bubbles were removed. The PDMS mixture was then poured onto feature‐side up silanised masters and degassed once more in a desiccator until all air bubbles were removed. PDMS‐covered masters were then cured in an oven for 4 h at 65˚C before cooling to room temperature. The PDMS mixture was then poured onto feature‐side up silanised masters and degassed once more in a desiccator until all air bubbles were removed, after which the PDMS‐covered masters were cured for 4 h at 65˚C in an oven before cooling to room temperature. Finally, the PDMS layer was carefully peeled off and cleaned in the oxygen plasma cleaner set to 100 W for 5 min to remove residual silane. PDMS stamps were cut into roughly 1 cm x 1 cm squares prior to use them.

### 2.5 Stamping of 3D collagen gels

3D collagen gels derived from rat tail collagen type I (Corning) were created on ice by making a solution containing a final concentration of 2 mg/ml collagen type I, 14.5 mM NaOH, 20 mM HEPES, 100 μg/ml fibronectin and 0.3% NaHCO_3_ (w/v) made up in Opti‐MEM supplemented with 10% FBS. A master mix of Opti‐MEM, FBS (Gibco), NaOH (Sigma‐Aldrich), HEPES, fibronectin (EMD Millipore Corp.) and NaHCO_3_ (Sigma‐Aldrich) was made up and added to collagen immediately prior to plating. Immediately following plating of the liquid collagen solution, the gel was polymerised at 37˚C in a sterile tissue culture incubator for at least 30 min after which time Opti‐MEM supplemented with 10% FBS and 1% penicillin‐streptomycin was added.

To enable visualisation of collagen hydrogel architecture and cell–ECM dynamics, Cy3 Mono‐reactive Dye (Cytiva) was used to stain 1 mg high‐concentration collagen. One day prior to staining collagen, dialysis buffers were made up consisting of 1L 0.1% acetic acid (Fisher Bioreagents) in dH_2_O (v/v) and 500 ml 0.1% acetic acid in PBS (calcium‐ and magnesium‐free, v/v). Dialysis buffers were left in the fridge overnight to cool to 4˚C. On ice, a 1 ml total mixture of high‐concentration collagen and 0.1 M NaHCO_3_ solution (pH 9.3 in Opti‐MEM, 0.2 μm sterile filtered) was made up according to the volume of collagen corresponding to 1 mg with the remainder added as NaHCO_3_ solution. Cy3 dye was first solubilised in NaHCO_3_ solution and vortexed for 1 min before being incubated on ice for 5 min. Collagen was then added and mix by pipetting slowly up and down at least 10 times. The Cy3 tube was covered in foil and incubated on a roller in the cold room for 1 h. Meanwhile, 8 kDa molecular weight cut‐off dialysis tubes were sterilised in 70% ethanol for 20 min on a roller before drying, while the cap was kept in sterile PBS. After 1‐h Cy3‐collagen incubation, the 1 ml mixture was added to dialysis tubes with a cap float and inverted for dialysis in the cold room on a rocker with chilled 500 ml 0.1% acetic acid in dH_2_O for 2 h, followed by dialysis in fresh 500 ml 0.1% acetic acid in dH_2_O overnight and one final dialysis in 500 ml 0.1% acetic acid in PBS for 2 h. Finally, Cy3‐stained collagen was transferred to a fresh Eppendorf on ice and covered with foil for storage at 4˚C.

**FIGURE 1:**
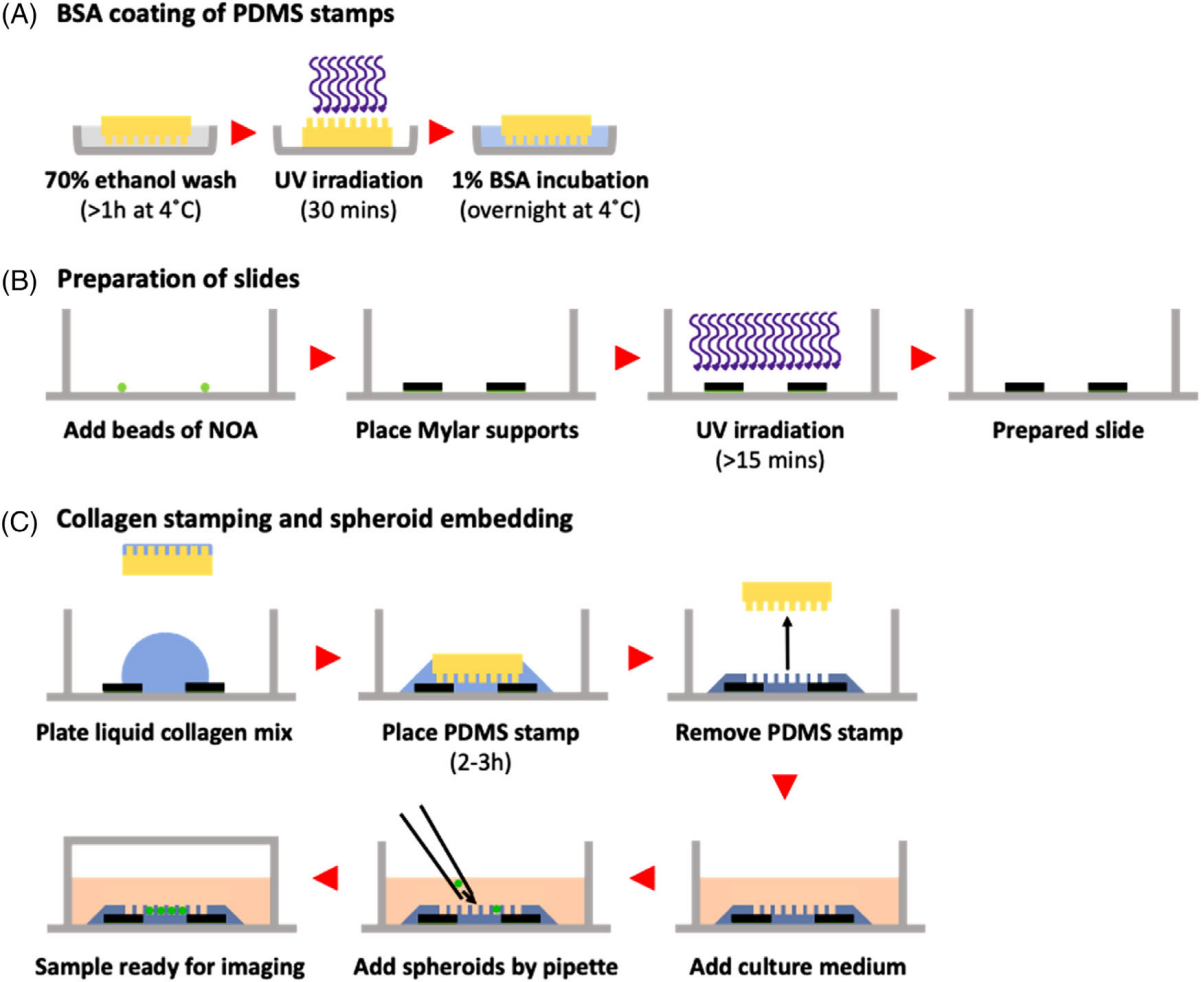
Schematic for preparation of stamped collagen hydrogels and embedding of spheroids. Schematic showing key steps and timings during preparation of stamped collagen hydrogels including (A) cleaning PDMS stamps (yellow) with 70% ethanol wash for at least 1 h followed by UV irradiation for 30 min and subsequent overnight BSA coating (light blue) of PDMS stamps at 4˚C, (B) preparation of slides with Mylar film support layers glued using Norland Optical Adhesive (NOA) which sets due to UV irradiation (shown in purple) and (C) PDMS stamping of liquid collagen (blue) plated onto prepared slide, removal of PDMS stamps and manual embedding of spheroids (green) following culture medium addition. NOA = Norland Optical Adhesive, BSA = bovine serum albumin, PDMS = polydimethylsiloxane.

Microfabricated PDMS stamps with micropillars were used to stamp well structures into 3D collagen gels, with an overview of the stamping process shown in Figure 1. Forty‐eight hours prior to sample preparation, as shown in Figure 1A, PDMS stamps were washed overnight in 70% ethanol then irradiated for 30 min with UV. A 1% BSA solution (Roche) was made up in Opti‐MEM (w/v) and 0.2 μm sterile‐filtered to reduce incidence of contamination. PDMS stamps were incubated feature‐side down in 1% BSA solution overnight in a 60 mM diameter tissue culture dish to reduce collagen gel attachment to PDMS surface and subsequent tearing of gels upon stamp removal. Prior to use during stamping, PDMS stamps were removed from 1% BSA solution and the feature‐side was rinsed once with Opti‐MEM.

To allow for defined distances between the surface of the slide and the bottom of the stamped collagen well, Mylar film (Radioshack Pro) of defined thickness was used as a support layer for stamps as summarised in Figure 1B. Prior to use, Mylar film was washed with 70% ethanol for 2 h on a rocker before drying and irradiating with UV for 30 min to reduce incidence of contamination. Small Mylar film squares of roughly 1.5 cm x 1.5 cm were cut and pairs of supports were placed using forceps onto ∼20 μl drops of Norland Optical Adhesive 81 (NOA; Norland Products Inc.). Positioning of Mylar supports was such to allow for PDMS stamps to rest on top of the supports and overlap the supports on both sides with an intersupport distance of between 0.5‐0.8 cm. Following positioning of supports, slides were irradiated in a UV box for a minimum of 15 min to allow the adhesive to set. If additional depth/height/distance is required (e.g. if investigating a highly invasive cell type), additional support layers may be stacked on top of each other.

After addition of Mylar supports, collagen gels were made up as above. Note that 120 μl liquid collagen gel was pipetted between Mylar supports and 50 μl pipetted onto the feature‐side of 1% BSA‐coated PDMS stamps. As detailed in Figure 1C, stamps were then inverted and placed feature‐side down on top of Mylar supports so that at least one row of pillars overlapped each support. To aid ease of spheroid identification under the microscope, stamps were positioned so that wells were micropatterned in an orthogonal grid orientation within the chamber. This means that adjacent spheroids can be found quickly at 90˚ orientations from other spheroids. Upon placement of collagen coated stamps onto Mylar supports, the sample was placed in a sterile tissue culture incubator and collagen was allowed to polymerise for 2–3 h at 37˚C. Following polymerisation, PDMS stamps were carefully removed using forceps by lifting vertically from the gels to increase accuracy of stamped well structures and 600 μl Opti‐MEM supplemented with 10% FBS and 1% penicillin‐streptomycin was added carefully on top of the stamped collagen gel before placing samples into a tissue culture incubator. PDMS stamps were placed in 70% ethanol on a roller to wash for reuse.

### 2.6 Generation of spheroids by hanging drop method

Multicellular tumour spheroids were formed using the hanging drop method. To generate drops, a methylcellulose (Sigma‐Aldrich) stock solution was generated by preheating 250 ml DMEM to 60˚C and using preheated DMEM to dissolve 6 g of autoclaved methylcellulose powder then stirring the solution for 20 min at room temperature. An additional 250 ml DMEM was added, and the solution stirred at 4˚C overnight. Methylcellulose solution was then cleared by centrifugation in 50 ml tubes at 5000 x g for 2 h at room temperature. Cleared methylcellulose solution was transferred to fresh 50 ml tubes and stored at 4˚C for use within 12 months. To generate spheroids, cells were trypsinised and counted as above. Cell number was optimised depending on cell line to size‐match spheroids to the diameter and depth of stamped collagen well features, with 400 cells (MCF7), 750 cells (HCC1954) and 1000 cells (NDF) found to produce 200 μm diameter spheroids. The required cell number per spheroid was added to make a 3:1 cell suspension:methylcellulose mixture with the cell suspension in full culture medium appropriate for the respective cell lines. Thirty litres of this mixture was pipetted onto the inner lid of a 10 cm^2^ tissue culture dish and 7 ml PBS was added to the bottom of the dish to prevent hanging drop evaporation. The lid was carefully inverted and placed onto the bottom such that 30 μl drops were hanging from the lid. The dish was incubated at 37˚C in a tissue culture incubator for 24–72 h until spheroids could be identified under a dissecting microscope.

### 2.7 Embedding of spheroids into collagen gel stamps

Following spheroid formation by hanging drop and stamping of collagen gels, spheroids were introduced to collagen gels in a class II, type A1 biosafety hood to reduce incidence of contamination. As detailed in Figure 1C, at least 4 spheroids were drawn up into an autoclaved gel loading tip and expelled onto the surface of the gel to disperse residual methylcellulose into Opti‐MEM covering the gel. Then, looking down the eyepiece of a dissecting microscope, spheroids were manually pipetted into the stamped wells by sucking or impelling with small bursts of media to pull/push spheroids towards wells and gently tapping spheroids into wells if required. Following spheroid introduction to wells, samples were left in the hood with their lids replaced for 15 min to allow for spheroid attachment to collagen gels and reduce likelihood of spheroids floating out from wells. Samples were then placed in a sterile tissue culture incubator for at least 30 min before imaging.

For staining with antibodies or dyes, medium was removed from spheroids embedded in stamped collagen gels by gently inverting slides and tapping out media, followed by one wash with PBS for 15 min on a rocker at room temperature. Samples remained covered by a foiled chamber throughout the staining protocols to reduce photobleaching of fluorophores. PBS was removed and samples were fixed by addition of 10% formalin with methanol (Sigma‐Aldrich) solution to samples for 2 h incubation at 37˚C. Following fixation, samples were washed thrice for 10 min each time in PBS followed by permeabilisation using 0.25% Triton X‐100 (Sigma‐Aldrich) in PBS (PBST, v/v) for 15 min on a rocker at room temperature. Samples were washed thrice for 10 min again before blocking with 5% BSA in PBST for at least 1 h at room temperature on a rocker. Following blocking, primary antibodies were added in a volume of 250 μl blocking buffer per gel (for 4‐chamber slides) for incubation overnight on a rocker at 4˚C. The following day, primary antibodies were removed, and samples were washed 3 times for 10 min before addition of appropriate secondary antibodies or dyes in 250 μl volume for 1 h incubation on a rocker at room temperature. Finally, samples were washed thrice for 10 min before final addition of 600 μl PBS and storage at 4˚C covered in foil until imaging.

## 3 MICROSCOPY

### 3.1 Phase contrast and epifluorescence microscopy

Spheroids were imaged using phase and epifluorescence microscopy on an EVOS FL Auto 2 microscope (Thermo Fisher Scientific) within an environmental chamber maintained at 37˚C. Phase contrast or epifluorescence images were captured with 10× air objectives using EVOS software (v2). For epifluorescence, a blue or green filtered LED light was used to excite GFP or mScarlet fluorophores respectively. Images were processed using FIJI.^13^ Quantification of ‘standard’ embedded spheroid invasion assays was carried out in FIJI and involved thresholding the central spheroid area and quantifying the area change over time relative to the initial time point.

### 3.2 Point‐scanning confocal microscopy

Images of fixed and live samples were captured on a Nikon A1R Ti inverted laser scanning confocal microscope within an Okolab chamber (Okolab S.R.L) maintained at 37˚C. Images were captured using a 20× ELWD Plan Fluor objective (NA 0.45) or 40× Plan Fluor WI objective (NA 1.25) with excitation wavelengths of 403 nm (diode laser), 488 nm (argon laser), 561 nm (diode laser) and 641 nm (diode laser) used. All confocal microscopy laser power and gain was optimised according to fluorophore intensity and detector saturation with a pinhole size of 44.7 μm to reduce detector saturation. NIS‐Elements imaging software (version 4.51.01) was used to capture images and files were saved in .nd2 format.

### 3.3 Imaging stamped collagen gel spheroid invasion assay and immunofluorescent stained spheroids

Stamped collagen gel spheroid invasion assays and immunofluorescent‐stained spheroids were imaged on a Nikon A1R Ti inverted laser scanning confocal microscope in a 37˚C Okolab chamber as above using Mylar support strips of 125 μm (MCF7 spheroids) or 250 μm (HCC1954 spheroids) to account for spheroid invasiveness. Identification of spheroids was accomplished using the epifluorescence mode with blue LED light with spheroids placed orthogonal to each other to aid identification. Images were captured using either a 20× ELWD Plan Fluor objective (NA 0.45) or 40× Plan Fluor WI objective (NA 1.25) for invasion assays and stained spheroids, respectively, with excitation wavelengths of 405 nm (diode laser), 488 nm (argon laser), 561 nm (diode laser) and 641 nm (diode laser) used as appropriate. To reduce sample photobleaching, phototoxicity and cell death, imaging parameters were optimised and set with lookup tables at 50% of their maximum value to allow for reduced laser power and increased scan speed. Invasion assays were imaged once every 24 h for a 96‐h period with the first time point occurring no later than 1 h after spheroid introduction into wells. The ND acquisition tab was used to store XY positioning of spheroids and parameters for imaging the Z‐plane. Each spheroid was imaged individually with 10 μm Z‐slices captured from the slide surface up to the upper surface of the collagen gel. Individual spheroid XY positions were updated at each time point to recentre spheroids. All imaging and positioning parameters were maintained throughout the 96‐h time‐course. For fixed samples, spheroids were imaged individually with 1 μm Z‐slices captured from 5 μm below the spheroid to 80 μm into the spheroid. Following imaging, live samples were incubated at 37 ˚C in a sterile tissue culture incubator.

### 3.4 SoRa microscopy of filopodia and time‐lapse imaging

For live imaging of filopodia in spheroids, samples were prepared as above with MCF7 GFP‐YAP/mScarlet LifeAct spheroids embedded in collagen gels with Mylar support strips of 75 μm. Samples were imaged live 24 h postembedding on a Nikon Eclipse Ti2 inverted microscope with Yokogawa CSU‐W1 SoRa confocal scanning unit (Yokogawa Electric Company) in a 37˚C Okolab chamber to enable optical capture of super‐resolution images and optimal sampling of filopodia. Samples were imaged with a CFI Apochromat LWD Lambda S 40× WI objective and using the SoRa disk with 50 μm pinhole diameter with excitation wavelengths of 488 and 561 nm used. For particle image velocimetry (PIV) time‐lapse analysis of spheroid‐influenced collagen ECM flow, samples were prepared as above with HCC1954 LifeAct‐GFP spheroids embedded in Cy3‐stained collagen gels with Mylar support strips of 75 μm. Samples were imaged live 24 h postembedding on a Nikon Eclipse Ti2 inverted microscope with Yokogawa CSU‐W1 SoRa confocal scanning unit in a 37 ˚C Okolab chamber as above. Samples were imaged with a CFI Apochromat LWD Lambda S 40× WI objective and W1 disk with 25 μm pinhole diameter with excitation wavelengths of 488 and 561 nm used. Images were captured using dual Photometrics Prime 95B sCMOS cameras through a 1× SoRa magnifier. Time‐lapse imaging was set to acquire Z‐stacks of 25 μm at slices of 0.5 μm through the central plane of each spheroid every 5 min for 30 min. Imaging parameters were optimised to enable rapid super‐resolution imaging of filopodia with 0.3 μm Z‐slices and 100 ms frame exposure while, to reduce photo‐toxicity, laser power and exposure time were set up with lookup tables set to 15% of maximum dynamic range. NDF spheroids were fixed and imaged with a CFI Apochromat LWD Lambda S 40× WI objective and W1 disk with 25 μm pinhole diameter with excitation wavelengths of 488 and 561 nm used. NIS‐Elements AR (version 5.21.03) was used for image acquisition; FIJI was used for postprocessing.

### 3.5 Multiphoton microscopy and ablation assay

To examine the role of ECM network tension in determining YAP protein subcellular localisation, multiphoton ECM ablation assays were carried out. MCF7 GFP‐YAP spheroids were embedded in Cy3‐stained collagen gels using 75 μm Mylar supports and imaged 24 h postembedding. Samples were imaged live on a Zeiss LSM 880 microscope (Carl Zeiss Ltd.) with Airy scan using an LD C‐Apochromat 40× WI Korr M27 objective (NA 1.1) using 1× zoom with excitation wavelengths of 488 nm (argon laser) and 561 nm (DPSS 561‐10 laser) used. For ECM ablation, ROIs were manually defined around the periphery of the spheroid at least 20 μm from the spheroid. A pulsed Chameleon Vision II TiSa laser (Coherent) tuned to 760 nm was used at 100% for 3 repeats at 15 ms per repeat. Spheroids were imaged in Z‐slices to generate a 144 μm region in Z composed of 72 Z‐slices per spheroid. Images were captured preablation and postablation, with time‐lapse imaging performed postablation with a temporal resolution of 10 min for 30 min total. Imaging parameters were optimised to reduce phototoxicity by setting laser power with lookup tables set to 50% of maximum dynamic range. Images were .czi format and image acquisition was completed using Zeiss ZEN (version 1.0) with postprocessing occurring using Fiji. Previous validation of ablation assays is shown in Tetley et al.^14^

### 3.6 Image analysis and statistical analysis

Particle image velocimetry (PIV) was performed as described in Yolland et al.^15^ with an open‐source MATLAB pipeline (https://github.com/stemarcotti/PIV). Briefly, the image at each time point is subdivided into overlapping squared portions of 5 μm (source size, 3 μm overlap). Each portion is then searched for in the next time point within a bigger surrounding region (search size, 10 μm) and best match is found by cross correlation (minimum correlation 0.3). A vector is defined between the position of each source size at the two subsequent time points, representing velocity of each feature. The raw field of obtained velocity vectors is then interpolated with a Gaussian kernel of size 50 μm and sigma 10 μm.

Alignment by Fourier Transform (AFT) was carried out as described in Marcotti et al.^16^ (https://github.com/OakesLab/AFT‐Alignment_by_Fourier_Transform). Orientation vectors were generated over 25 × 25 pixel windows across the image (overlap 50%) to obtain a vector field representing local fibre orientation.

Experiments are typically composed of a minimum of 4 spheroids per condition with 3 independent experimental repeats. We find that this allows for robust statistical analyses that are highly reproducible both within and between experimental repeats, with typical inter‐ and intraexperimental differences of ±5% with respect to different phenotypic endpoints analysed.

## 4 RESULTS

### 4.1 Imaging of embedded multicellular breast cancer spheroid invasion and spheroid immunostaining

**FIGURE 2:**
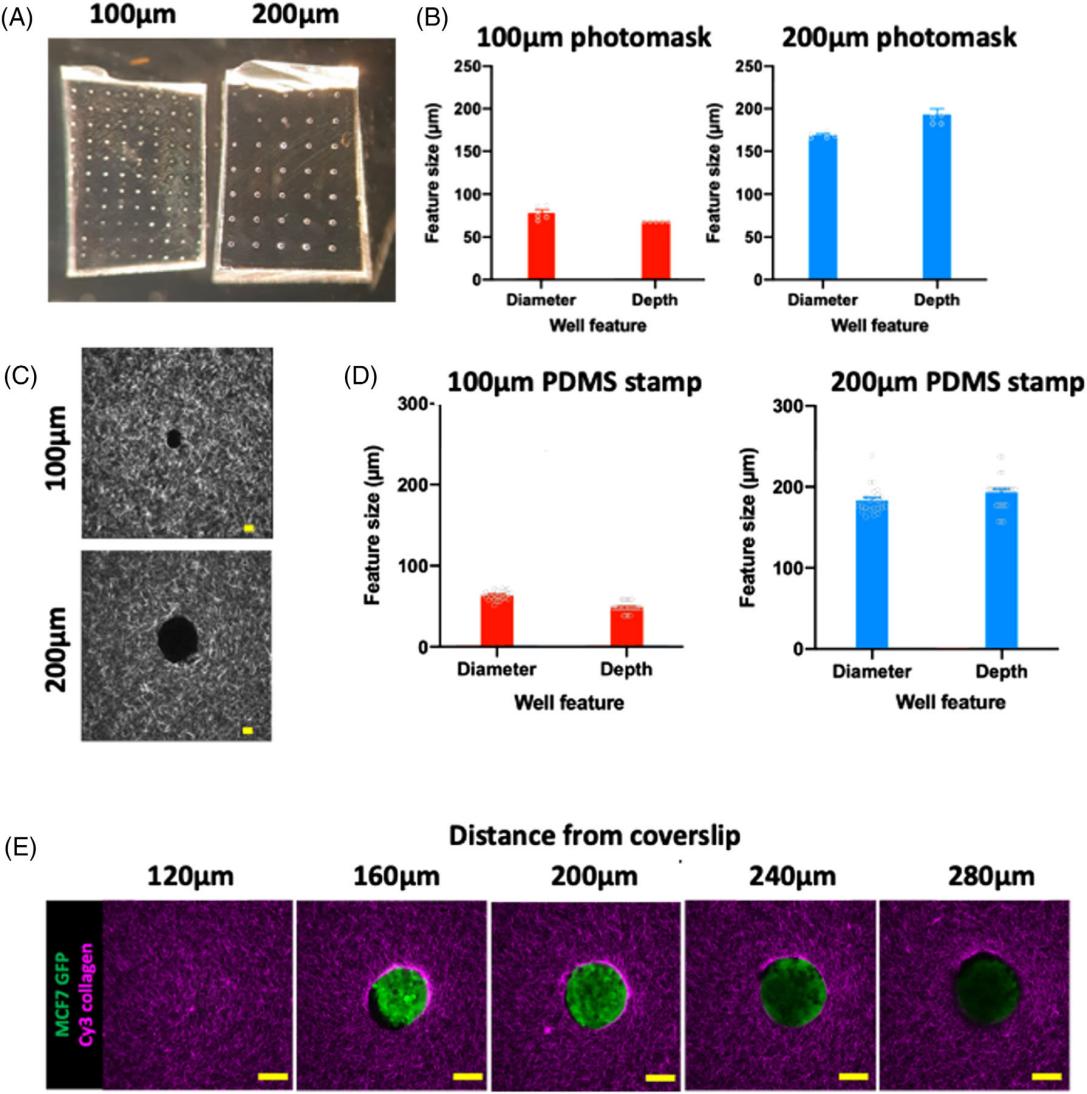
Characterisation of SU8‐2100 masters, PDMS stamps and stamped collagen wells. (A) 6× magnified images of PDMS stamps generated from 100 μm diameter/500 μm pitch (left) and 200 μm diameter/950 μm pitch (right) SU8‐2100 masters. (B) Characterisation of two different SU8‐2100 master templates (100 μm diameter, left, and 200 μm diameter, right) in terms of well diameter and well depth. Data presented as means ± SEM with individual data points shown in grey. Representative of 5 distinct regions from each SU8‐2100 master. (C) Example confocal images of Cy3‐stained stamped collagen wells (greyscale) with images shown from 100 μm (top) and 200 μm (bottom) PDMS stamps. Scale bars represent 50 μm. D) Quantification of stamped collagen well diameters and depths when stamped with 100 μm (red) and 200 μm (blue) diameter PDMS stamps. Data presented as means ± SEM with individual data points shown in grey. Representative of at least 5 different wells each from three different experimental repeats. (E) Representative Z‐slice series through a Cy3‐stained stamped collagen well (magenta) with embedded MCF7 GFP spheroid (green) at 40 μm intervals. Scale bars represent 100 μm. Images representative of minimum 4 spheroids per condition and three independent repeats.

For characterisation, different PDMS stamp types were generated that had different sizes and pitch of features with examples shown of 100 μm diameter and 500 μm pitch on the left and 200 μm diameter and 950 μm pitch on the right. The PDMS stamps (Figure 2A) were generated from SU‐8 2100 master templates which we characterised the diameter and depth of, with feature sizes for both 100 μm (left) and 200 μm (right) diameter master templates (Figure 2B) verified. Characterisation of these PDMS stamps was important to better understand whether specifications patterned onto SU‐8 2100 master templates were faithfully conveyed to the resulting PDMS stamps. Similarly, it was important to also characterise how well these structures conveyed the desired feature size when stamping into collagen gels. Representative images captured with an inverted point‐scanning confocal microscope show example stamped collagen well structures with quantification of well diameter and depth shown for both PDMS stamp types imaged 50 μm from the bottom of the well (Figure 2C and D), together with an example series of Z‐slices running through a stamped collagen well with an embedded MCF7 GFP spheroid (Figure 2E). As we originally developed this method as an invasion assay, our choice of MCF7 and HCC1954 human breast cancer cell lines was based on two factors: (1) their propensity to form multicellular tumour spheroids by hanging drop and (2) MCF7 spheroids are noninvasive whereas HCC1954 undergo invasion, providing means to assess both phenotypes within the same reproducible platform.

**FIGURE 3:**
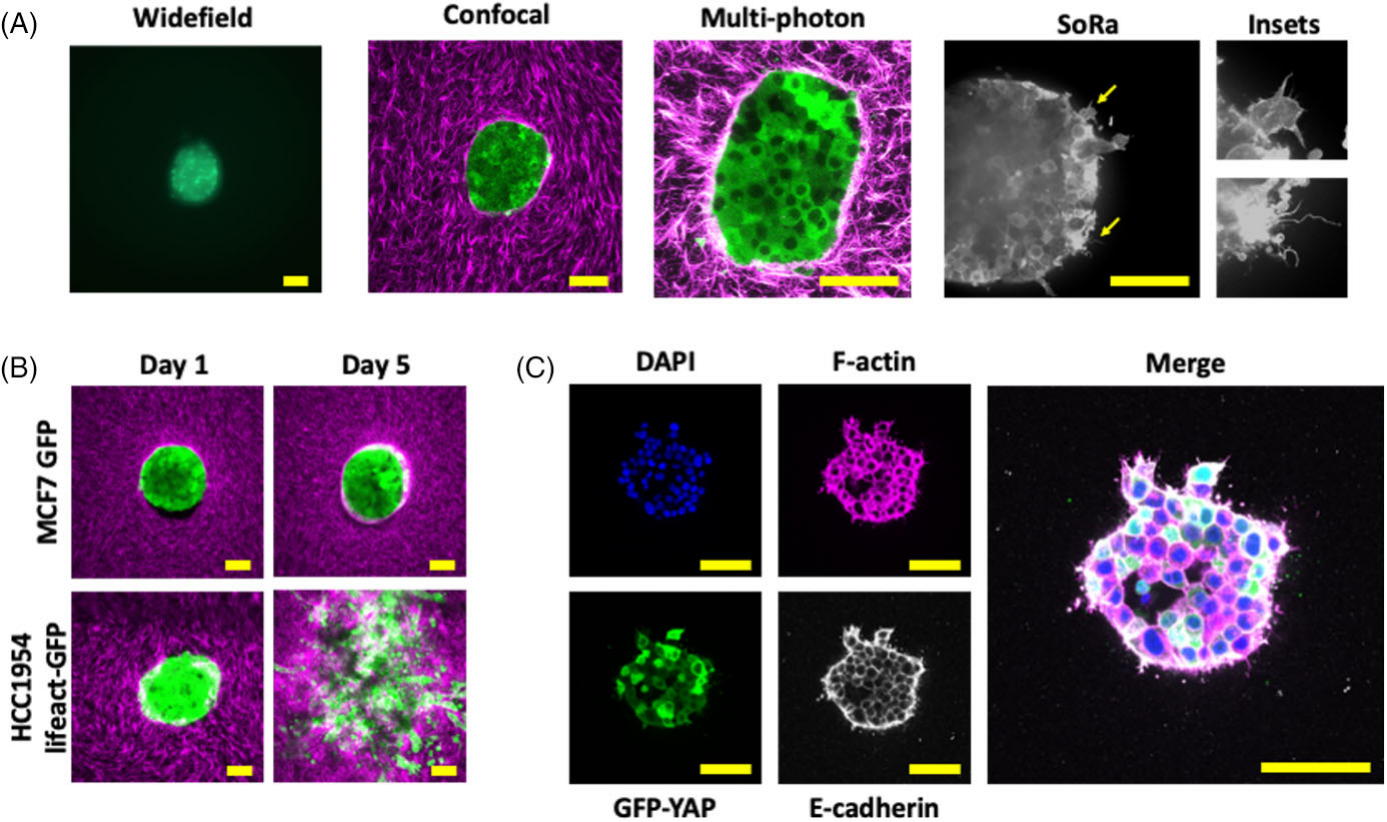
Imaging breast cancer spheroids embedded in stamped collagen gels. (A) Single Z‐slice representative images of different MCF7 GFP spheroids imaged with (left to right) 10× widefield epifluorescence, 20× point‐scanning confocal, 40× multiphoton and, 40× super‐resolution SoRa systems (with zoomed insets showing fine structure). Green = GFP, magenta = Cy3‐stained collagen, greyscale = lifeact‐mScarlet. (B) Representative MIPs at Day 1 (left) and Day 5 (right) invasion time points with noninvasive MCF7 GFP (top) and invasive HCC1954 lifeact‐GFP (bottom) spheroids (both green) invading into Cy3‐stained stamped collagen gels (magenta). Images representative of minimum 12 spheroids per cell line and three independent repeats. (C) Representative images of staining spheroids in stamped collagen gels with dyes and antibodies. Embedded MCF7 GFP‐YAP (green) spheroid shown stained with DAPI (blue), phalloidin‐567 (magenta) and E‐cadherin with AlexaFluor 647 secondary (grey), with merge shown on left. Images representative of minimum 12 and three independent repeats. All scale bars represent 100 μm. SoRa = super‐resolution by optical reassignment, MIP = maximum intensity projection.

To determine the suitability of these 3D samples for commonly used microscopes, images were captured using widefield epifluorescence, point‐scanning confocal, multiphoton or super‐resolution spinning disk microscopes (representative images shown in Figure 3A). These images were captured within the central region of each spheroid ∼75 μm from the bottom of the spheroid using a variety of objective lenses from 10× in the widefield epifluorescence image to 40× with the SoRa images. In general, this sample preparation method is suitable for imaging spheroids with any inverted light microscope in which Z‐distance is a barrier to successful imaging. Inset images from the SoRa microscope show the ability to capture super‐resolution images of fine actin‐based protrusions at the spheroid periphery such as filopodia. By adjusting the thickness of the Mylar film used, it was possible to bring spheroids within the working distance of most objective lenses.

Figure 3B shows representative images from Day 1 and Day 5 of spheroid invasion assays using noninvasive MCF7 GFP spheroids (top row) and invasive HCC1954 lifeact‐GFP spheroids (bottom row). During this invasion assay, a ∼400 μm Z‐stack was captured at 10 μm Z‐intervals of each spheroid every 24 h for 5 days using an inverted point‐scanning confocal microscope with 20× objective, with optimised imaging parameters to reduce photo‐toxicity. Displayed images are maximum intensity projections (MIPs) of a representative 30 μm region. Notably, use of a 20× air objective enabled images of the entire gel to be captured from bottom to top. Finally, these spheroids were also successfully stained with dyes and antibodies such as DAPI (blue), phalloidin (magenta) and an E‐cadherin primary antibody (grey) (Figure 3C). E‐cadherin was chosen as a marker to both validate the immunostaining technique and to understand the integrity of cell‐cell adhesions within spheroids following placement within these collagen wells. These images were captured with an inverted point‐scanning confocal microscope ∼40 μm from the base of the spheroid. The E‐cadherin antibody staining reveals slight antibody penetration deficiency evidenced by an apparent gradient in the staining pattern. This is a phenomenon typically seen when staining tumour spheroids and is dependent on both the spheroids size and density. Thus, embedding spheroids in stamped collagen hydrogels provides a versatile platform for high‐/super‐resolution imaging of otherwise challenging 3D structures.

### 4.2 Applications for study of cell–ECM interactions using spheroids embedded in micropatterned collagen hydrogels

**FIGURE 4:**
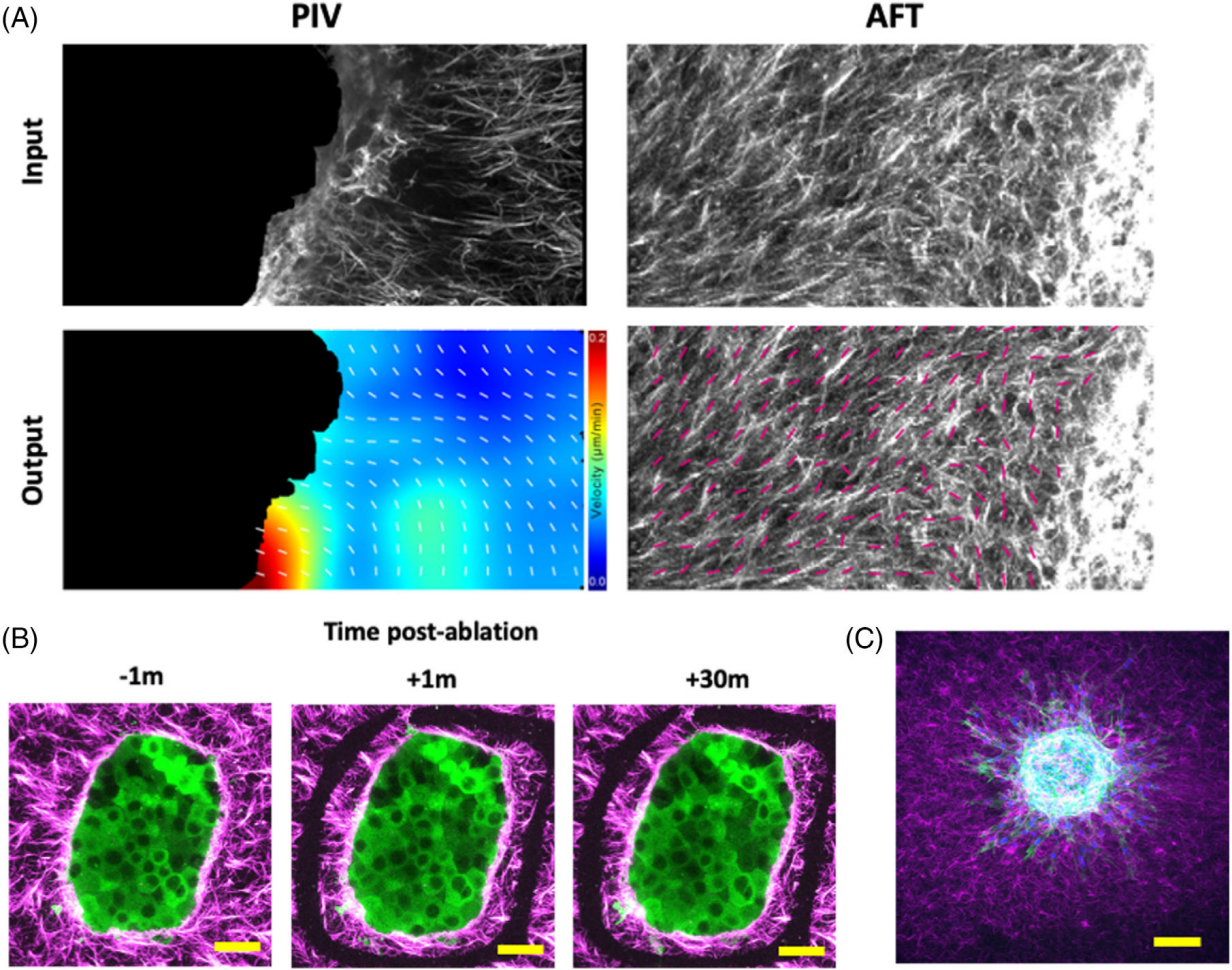
Using spheroids embedded in stamped collagen gels to examine cell–ECM interactions. (A) Representative data presented from particle image velocimetry (PIV, left) and Alignment by Fourier Transform (AFT, right) analyses run on Cy3‐stained collagen datasets. Input images are shown at the top, with analysis output images for the respective analysis approaches shown below. For PIV, magnitude of velocity is shown by interpolated colours, with black representing the masked region occupied by the spheroid. For AFT, magenta arrows show mean direction of local collagen fibres. (B) Representative MIPs of MCF7 GFP‐YAP (green) spheroid embedded in Cy3‐stained, stamped collagen well (magenta) pre‐ (left), 1 min and 30 min postablation (middle/right respectively). Ablated region peripheral to spheroid can be seen as black regions missing any magenta signal. (C) Representative MIP of a normal dermal fibroblast (NDF) organoid embedded in Cy3‐stained collagen (magenta). NDF organoid stained with DAPI (blue) and phalloidin‐488 (green). All scale bars represent 100 μm. All images representative of minimum 4 spheroids per condition and three independent repeats. MIP = maximum intensity projection.

While this method was initially developed with the intention of imaging spheroid invasion assays at high‐/super‐resolution, other applications were also tested to expand the repertoire of this method into the study of cell–ECM interactions. Figure 4A shows representative outputs from particle image velocimetry (PIV, left) and Alignment by Fourier Transform (AFT), which can be used to measure collagen matrix movement and alignment, respectively. Input data into PIV analyses were 5‐min time lapses at 30 s intervals captured using the SoRa microscope ∼75 μm deep into the gel with a 40× water‐immersion (WI) objective. Multiphoton microscopy imaging was further used to ablate collagen matrices around the spheroid periphery to examine the effect of releasing ECM tension on intracellular protein localisation (specifically, YAP‐GFP in MCF7 cells; Figure 4B). Samples were imaged live using a Zeiss LSM 880 microscope with Airy scan using a 40× WI objective, with a 144 μm region from the base of the spheroid (75 μm deep) towards the top of the spheroid imaged. Ablation was successfully achieved with a pulsed TiSa laser tuned to 760 nm and used at 100% power for 3 × 15 ms repeats within a defined ROI following the initial time point and with time‐lapse imaging following this every 10 min for 30 min. Following MP ablation of the surrounding ECM as shown by black regions corresponding to designated ROIs in Figure 4B and previously demonstrated Tetley et al.,^14^ we observed subcellular changes in YAP protein localisation 30 min postablation. Finally, this approach was adapted to permit imaging of primary normal dermal fibroblasts (NDFs) forming cell–ECM interactions (Figure 4C). The NDF organoid was embedded in 200 μm diameter stamped collagen wells and imaged using the 40× water immersion objective on the SoRa microscope ∼150 μm into the sample. In conclusion, this sample preparation technique enables robust and reproducible imaging for study of cell–ECM interactions.

## 5 DISCUSSION

The method presented here provides a robust and repeatable strategy for high‐/super‐resolution imaging of multicellular spheroids embedded within collagen hydrogels. We were able to reproducibly use this technique to stain spheroids, accomplish rapid live imaging, examine cancer and fibroblast cell invasion/migration from spheroids and leverage advanced image analysis approaches to study cell–ECM interactions. Although we focused our analysis on Ibidi chamber slides with inverted microscopy, this method can be applied to any tissue‐culture treated or collagen‐coated surface for either inverted or upright optical microscopy. We provide a ‘quick reference’ protocol for this approach in Supplementary File S1.

There are several benefits of this method compared to previous approaches. Most notably, this method enables for precision positioning of spheroids in 3D collagen hydrogels in contrast to standard spheroid embedding which typically results in inconsistent placement in the Z‐axis. The main benefit of this precise placement in 3D space is consistent high‐resolution imaging using inverted microscopy that partly defrays issues experienced when using high‐power/numerical aperture objective lenses on these samples. Consistency of imaging is achieved by uniformity in sample distance from the coverslip by virtue of Mylar support layers resulting in uniform positioning of collagen well bottoms. Precision in XY placement also allows for known interspheroid distances, aiding location of spheroids during imaging. In comparison to the method laid out by Piotrowski‐Daspit and Nelson,^12^ there are notable differences that distinguish our method such as dimensions of stamped features and addition of preformed spheroids into stamped wells, with the latter being particularly important when deploying our method primarily to monitor cancer cell invasion using high‐resolution imaging. Furthermore, placement of multiple spheroids within the same gel allows for a reduction in variation often seen between different wells during standard spheroid invasion assays, although this comes with the caveat that spheroids in the same environment may influence each other. This platform is also amenable to studying the effect of variable biophysical characteristics such as matrix density or stiffness using crosslinkers such as ribose

More intricate or varied PDMS stamp designs such as different diameter, shape or depth of features would enable this approach to be further adapted to different sizes or types of samples depending on the biological question being addressed. Further adaptations to this model could be applied to enable higher throughput. Manual addition of spheroids by pipetting is a bottleneck in transitioning to high content use of this method. Semi‐automated spheroid placement could be accomplished via development of a size‐matched centrifugal fluidic device to funnel spheroids from a 96‐well plate to a stamped collagen gel in similar fashion to Weisler et al.^17^ Microfluidic robots or other microfluidic delivery systems could be used to automate placement and positional registration of spheroids within a spatially defined stamped gel for time‐course imaging or for AI‐mediated automated spheroid detection and analysis.

Scaling up the assay for drug discovery or drug screening has the potential to be a very powerful tool with some additional improvements. Current addition of compounds is limited to exposure of all spheroids in a single gel to one condition. Pretreatment of spheroids within hanging drop or an intermediate phase prior to addition to the stamped collagen gel would remove this constraint and allow for automated pretreatment of spheroids with a plethora of compounds before addition to the stamped gel. Modifications could also be made to increase the cellular and microenvironment complexity. For example, stem cell‐ and patient‐derived organoids more closely resemble the in vivo tumour environment and have potential for developing personalised treatments or therapies. While using cell line‐generated spheroids is a strength due to the flexibility, ease of use and generation, more physiologically relevant organoids could also be used in these 3D imaging chambers in future to study longitudinal behaviour at high resolution. Notably, use of liquid handling robotics could allow for this assay to be scaled up and largely automated with the ability to stamp collagen wells in many different sample holders lending itself to flexibly scaling up.

Microfluidics and 3D printing would also enable custom sample holders to be designed and produced for adding biological and mechanical complexity to experimental systems. For instance, Ko et al. designed a ‘spheroid on a chip’ spheroid‐HUVEC coculture system to study angiogenesis within spheroids^18^ while Huang et al. used a microfluidic device to examine the impact of increased interstitial flow on spheroid cell‐cell junctions.^19^ Other similar devices have been designed to study spheroid invasive response to dynamic epidermal growth factor (EGF) gradients^20^ and to monitor spheroid metabolism over multiday time periods.^21^ Furthermore, the use of ‘bio‐inks’ permits printing of various structures with filament‐based techniques at resolutions of 100 μm into collagen, Matrigel or decellularised matrix.^22^ Plane‐based techniques are best suited to print higher resolution 3D structures, although these approaches are limited by available bio‐inks and large volume requirements making them relatively expensive. In general, bio‐inks allow for inclusion of cells during dispersal which can facilitate even distribution of cells throughout the 3D printed structure, generating an effective coculture system with fibroblasts, endothelial and/or immune cells. Future use of 3D printing and bio‐inks to generate collagen scaffolds with variable dimensions and flexibility for precision 3D imaging may negate the requirement for the PDMS stamps used here and provide greater flexibility of experimental design. Combining microfluidic design and liquid handling robotics would further enable this assay to be scaled up for compound screening purposes in future.

## Supporting information

Supplementary File S1: ‘Quick guide’ step‐by‐step protocol to prepare model
