# Supplementary material for: A method for reproducible high‐resolution imaging of 3D cancer cell spheroids
Source: J Microsc. 2023 Jan 26;291(1):30–42. doi: 10.1111/jmi.13169 (PMC10953429; doi:10.1111/jmi.13169)
Supplement: Supplementary file 1 — Supplementary File S1: ‘Quick guide’ step‐by‐step protocol to prepare model [file JMI-291-30-s001.docx]

**Quick Reference Protocol**

Design and Microfabrication of SU-8 2100 Master Wafers

1. Custom design and print photomasks on film substrate to well diameter specifications (below protocol for 200µm thick master).
2. Clean silicon wafer with oxygen plasma cleaner at 100% for 5 mins and dehydrate at 200˚C for 30 mins. All following microfabrication steps should be carried out in dust-free, ventilated microfabrication lab.
3. Spin-coat wafer with 10mL SU-8 2100 with speed determining wafer thickness and ultimately stamped collagen well depth (in our use case, 2440 x g generated a 200µm thick layer)
4. Soft bake wafers at 65˚C for 5 mins, then 95˚C for 15mins.
5. Using a bulldog clip, make up a ‘sandwich’ of, in order, one glass layer, the SU-8 2100 silicon wafer, the desired photomask with micropattern (print-side in contact with SU-8 2100) and one quartz layer.
6. Place sandwich in UV exposure masking system photomask-side up (i.e. UV exposure through quartz layer and photomask onto SU-8 2100 surface) and continuously expose to UV at 35 mW/cm^2^ for 35 seconds.
7. Post-exposure, bake master wafer for 5 mins at 65˚C, then for 15 mins at 95˚C.
8. Submerse in SU-8 developer for 20 mins on a rocker to remove uncured SU8-2100.
9. Dry and hard bake masters for 1 min at 130˚C.
10. Store for use during microfabrication of PDMS stamps.

*Microfabrication of PDMS Stamps*

1. Expose SU8-2100 masters feature-side up in oxygen plasma cleaner set to 100% power for 10 mins.
2. Place masters in desiccator with trichloro(1H,1H,2H,2H-perfluorooctyl)silane for 1 hour to silanise surface of masters (aids in removal of PDMS).
3. Wash masters twice with acetone and once with isopropanol before drying with N_2_ flow.
4. Mix 10:1 (w/w) Sylgard 184 Elastomer (PDMS):curing agent and degas in desiccator to remove air bubbles.
5. In the desiccator, pour PDMS mixture onto feature-side of masters in a dish and degas until all air bubbles are removed. Take care to gently reintroduce air pressure to keep masters stable.
6. Cure PDMS-covered masters for 4 hours at 65˚C then allow to cool to room temperature.
7. Carefully peel off PDMS layer and clean in oxygen plasma clear at 100% power for 5 minutes to remove residual silane.
8. Cut PDMS stamps into desired dimensions for collagen gel stamping and store until use.

*Stamping of Well Structures and Embedding Spheroids into 3D Collagen Hydrogels*

Note: In this protocol, we stamped collagen gels into Ibidi 4-chamber slides. This protocol is likely suitable for most collagen gel recipes and for alternative sample holders such as dishes and chamber slides with removable slides. This section of the protocol should be carried out in a tissue culture hood to limit contamination.

1. Prepare multicellular tumour spheroids by hanging drop prior to sample preparation. Spheroids must be size matched against the diameter/depth of collagen wells by altering cell number per spheroid.
2. At least 24 hours prior to sample preparation, wash PDMS stamps in 70% ethanol for 1 hour then irradiate for 30 mins under a UV lamp.
3. Make up a 1% BSA solution (w/v) in OptiMEM and sterile filter with 0.2µm filter.
4. Incubate PDMS stamps feature-side down in BSA solution overnight on a rocker at 4˚C.
5. Prior to sample prep, cut Mylar film of chosen thickness (this defines the distance from slide surface to bottom of spheroid) into roughly 5x5cm squares and wash in 70% ethanol for 2 hours before drying and irradiating with UV for 30 mins.
6. On the day of sample prep, cut Mylar film into smaller 1.5x1.5cm squares and place two pieces using forceps onto two 20µL drops of Norland Optical Adhesive (NOA) on the slide spaced close enough for PDMS stamps to overlap both pieces of film (roughly 0.5-0.8cm).
7. Place the slide with Mylar film/NOA under UV lamp for at least 10 minutes.
8. Prepare collagen mixture on ice but do not initiate polymerisation.
9. Remove PDMS stamps from 1% BSA solution and wash once with PBS.
10. Retrieve slide with adhered Mylar film.
11. Initiate collagen gel polymerisation and add 50µL of liquid collagen mixture to PDMS stamp feature surface and 120µL to slide surface between Mylar supports.
12. Invert PDMS stamp coated with liquid collagen mixture and place feature-side down overlapping both Mylar supports.
13. Place slide into tissue culture incubator at 37˚C for 2 hours to allow collagen gel to polymerise fully.
14. Following polymerisation, add cell culture media around PDMS stamp to aid in removal. Use forceps to lift PDMS stamp directly upwards.
15. Place stamp in 70% ethanol and leave on rocker at 4˚C until needed again (repeat from step 1).
16. Spheroids can now be added to stamped collagen hydrogels by manually pipetting onto the hydrogel surface and using the pipette to gently draw/push the spheroid towards stamped wells using bursts of media. Spheroids can be gently ‘tapped’ into the well with the pipette tip if necessary. We used a dissecting scope in a class II, type A1 biosafety hood to aid spheroid addition.
17. Samples should be covered and left for 10 minutes to aid spheroid-ECM adhesion before moving to a tissue culture incubator for at least 30 minutes prior to use.

*Staining of Fixed Spheroids with Antibodies and Dyes in Stamped Collagen Gels*

1. Remove media from embedded spheroids by gently inverted slides and tapping out media.
2. Wash once with PBS for 10 mins on a rocker at room temperature.
3. Remove PBS and add 10% formalin solution for 2 hours in a tissue culture incubator at 37˚C.
4. Wash sample thrice for 10 mins with PBS on a rocker at room temperature.
5. Permeabilise with 0.25% Triton X-100 in PBS (PBST, v/v) for 30 mins on a rocker at room temperature.
6. Wash sample thrice for 10 mins with PBS on a rocker at room temperature.
7. Block with 5% BSA in PBST for at least one hour on a rocker at room temperature.
8. If immunostaining, incubate with primary antibody overnight at 4˚C on rocker.
9. Wash sample thrice for 10 mins with PBS on a rocker at room temperature.
10. Incubate with secondary antibody or dye for one hour on a rocker at room temperature.
11. Wash sample thrice for 10 mins with PBS on a rocker at room temperature and store in fridge prior to imaging.
